# *BIRC5* Gene Polymorphisms Are Associated with a Higher Stage of Local and Regional Disease in Oral and Oropharyngeal Squamous Cell Carcinomas

**DOI:** 10.3390/ijms242417490

**Published:** 2023-12-14

**Authors:** Ivan Mumlek, Petar Ozretić, Maja Sabol, Matko Leović, Ljubica Glavaš-Obrovac, Dinko Leović, Vesna Musani

**Affiliations:** 1 Department of Maxillofacial and Oral Surgery, University Hospital Centre Osijek, Josipa Huttlera 4, 31000 Osijek, Croatia; imumlek@gmail.com; 2 Faculty of Medicine, Josip Juraj Strossmayer University of Osijek, Josipa Huttlera 4, 31000 Osijek, Croatia; lgobrovac@mefos.hr; 3 Laboratory for Hereditary Cancer, Division of Molecular Medicine, Ruđer Bošković Institute, Bijenička Cesta 54, 10000 Zagreb, Croatia; pozretic@irb.hr (P.O.); maja.sabol@irb.hr (M.S.); 4 Clinical Hospital Centre Zagreb, Kišpatićeva 12, 10000 Zagreb, Croatia; matko.leovic@gmail.com; 5 Maxillofacial Surgery Unit, Department of Otorhinolaryngology and Head and Neck Surgery, Clinical Hospital Centre Zagreb, Kišpatićeva Ulica 12, 10000 Zagreb, Croatia

**Keywords:** oral squamous cell carcinoma, oropharyngeal squamous cell carcinoma, survivin, *BIRC5*, biomarkers

## Abstract

Oral squamous cell carcinoma (OSCC) and oropharyngeal squamous cell carcinoma (OPSCC) are the most common types of cancers in the head and neck region (HNSCC). Despite very aggressive treatment modalities, the five-year survival rate has not changed for decades and is still around 60%. The search for potential specific biomarkers of aggressiveness or outcome indicators could be of great benefit in improving the treatment of these patients. One of the potential biomarkers is survivin, the protein product of the *BIRC5* gene. In this study, we investigated the occurrence of *BIRC5* gene polymorphisms in 48 patients with OSCC and OPSCC compared with healthy controls. A total of 18 polymorphisms were found, 11 of which occurred in HNSCC with a minor allele frequency (MAF) of more than 5%. Five polymorphisms (rs3764383, rs9904341, rs2071214, rs2239680, rs2661694) were significantly associated with tumor size, tumor stage, and advanced regional disease, but had no impact on survival.

## 1. Introduction

Squamous cell carcinomas of the head and neck (HNSCC) originate from the mucosa of the upper aerodigestive tract, with the most common sites being in the oral cavity, oropharynx, and larynx, and less frequently in the hypopharynx, nasal cavity, and sinuses. Of these, oral squamous cell carcinoma (OSCC) and oropharyngeal squamous cell carcinoma (OPSCC) accounted for ~476,000 new cases and ~226,000 deaths in 2020 [1]. The majority of the OSCC cases worldwide are diagnosed in Asia, with the highest incidence in southeastern countries such as Sri Lanka, India, and Pakistan [1].

The main etiological factors are the consumption of alcohol and tobacco products, while HPV infection has become the leading cause of oropharyngeal cancer over the last decade in addition to the aforementioned factors [2].

Mutations in genes can lead to the development of cancer in the oral cavity and oropharynx. However, there is no specific gene that is responsible for oral or oropharyngeal squamous cell carcinomas. Environmental factors, such as smoking, alcohol abuse, radiation exposure, and viral infections, can activate proto-oncogenes (*RAS*, *MYC*, *EGFR*) or inhibit tumor suppressor genes (*TP53*, *RB1*, *CDKN2A*), potentially raising the risk of cancer in the oral and oropharyngeal regions [3].

Patient management relies on traditional histological parameters such as TNM staging and tumor grading. The treatment of choice for tumors of the oral cavity and oropharynx is primary surgery or primary chemoradiotherapy [4]. In recent years, immunotherapy has also been used to treat locoregionally advanced and metastatic disease [5]. Although the majority of patients with early-stage HNSCC can be cured with surgery or radiation, patients with aggressive disease and those with locally advanced stages, which account for two-thirds of new diagnoses, are more likely to recur (50% 5-year overall survival) [5].

However, ongoing research has explored novel molecular and cellular markers to improve patient care and survival rates. Therefore, there is a need for new biomarkers that can describe the diversity of cancers and provide the ability to categorize patients for tailored treatment approaches [6].

One of the potential targets is survivin, the protein product of the *BIRC5* gene. Survivin is a member of the inhibitor of apoptosis family. While survivin is present during fetal development in humans, it is normally absent in adult cells. Previous research has shown that survivin is remarkably abundant in most cancer cells. Therefore, it is a promising candidate for anti-cancer drugs and a potential tool for prognosis [7].

The *BIRC5* gene covers 14.7 kb of DNA at the telomeric end of chromosome 17q25 and consists of four exons separated by three introns. Its sequence encodes the protein survivin, which has 142 amino acids and a size of 16.5 kDa [8,9]. All existing survivin isoforms contain only one of the characteristic N-terminal BIR (Baculovirus IAP Repeat) domains, while the extended carboxy-terminal α-helix is replaced by the IAP characteristic “RING-finger” domain. The BIR domain plays an important role in the antiapoptotic function of survivin, while the amphipathic α-helix influences tubulin structures [9].

Increased *BIRC5* mRNA expression was detected in samples from patients with OSCC compared to peritumoral or normal tissues [10]. However, this increase alone was not sufficient to promote tumor progression in oral squamous cell carcinoma. Nevertheless, nuclear expression of survivin was observed to be associated with tumor stage and differentiation grade [10]. In OCSS, treatment with YM155 resulted in reduced survivin levels and an increased rate of apoptosis [11].

There is evidence that several single-nucleotide polymorphisms (SNPs) in the *BIRC5* promoter and the 3′UTR region have some influence on survivin expression [12,13]. The role of *BIRC5* polymorphisms has been studied in various cancers, and several polymorphisms have been associated with susceptibility [14,15,16], survival [16,17,18], and age of onset [19,20].

The aim of this study was to investigate *BIRC5* polymorphisms in oral and oropharyngeal squamous cell carcinomas and to correlate them with clinicopathological data.

## 2. Results

### 2.1. Subject Data and Classification

Forty-eight HNSCC patients and seventy-four healthy controls were selected for this study. Blood and tumor tissue were collected from each patient. The clinical data of the patients are presented in Table 1.

### 2.2. BIRC5 Polymorphisms in Patients with HNSCC and Healthy Controls

A total of eighteen polymorphisms were found in the constitutional DNA of 48 HNSCC patients and 74 healthy controls (Table 2). The vast majority of the polymorphisms found were located in the *BIRC5* promoter and in the 3′UTR region. All polymorphisms were in Hardy–Weinberg equilibrium. The polymorphisms rs143396310 (c.-1458C>T), rs17878731 (c.-267G>A), and rs17882627 (c.*104G>A) were found only in the controls, while rs772161908 (c.221+1199G>A) and rs761692199 (c.*111G>C) were found only in HNSCC samples. There were no significant differences in the distribution of genotypes or alleles between HNSCC samples and controls for any of the variants. However, for the polymorphism rs17887126 (c.-235G>A), slightly significantly higher frequencies of the heterozygous GA genotype and the minor A allele were observed in the HNSCC samples (*p* = 0.056 and *p* = 0.060, respectively).

### 2.3. Linkage Disequilibrium

**Figure 1:**
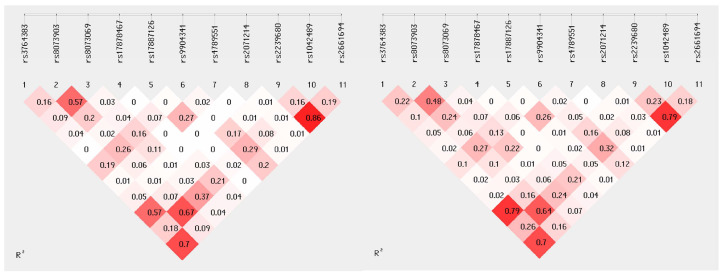
Pairwise linkage disequilibrium (LD) of eleven *BIRC5* polymorphisms in healthy controls and HNSCC samples. The position of each sequence variant along the *BIRC5* gene is indicated relative to the real nucleotide position. The number in each diamond indicates the intensity of LD (R^2^) between the respective pairs of SNPs. The strength of LD is also represented by shades of red (0 [white] < R^2^ < 1 [red]).

Eleven SNPs with a minor allele frequency (MAF) >5% in HNSCC samples were selected for linkage disequilibrium analysis. The analysis showed that there was no difference in the number of polymorphisms in linkage disequilibrium (LD) between HNSCC samples and controls (Figure 1). Both HNSCC and healthy controls showed the highest nonrandom association of alleles between polymorphisms rs2239680 and rs2661694. In addition, the most significant increase in pairwise LD intensity was observed in SCC samples compared to controls for rs3764383 and rs2239680.

### 2.4. Association of BIRC5 Polymorphisms with Clinicopathological Variables in HNSCC Patients

Clinicopathological findings (age at diagnosis, gender, smoking status, tumor site, tumor size, clinical and pathological lymph node status, clinical and pathological TNM status, Broders status, and survival) were compared with eleven SNPs with a MAF > 5%.

*BIRC5* polymorphisms were not associated with age and survival and only weakly associated with location and Broders status (Supplementary Table S1). Four polymorphisms (rs8073903, rs8073069, rs17878467, and rs1042489) were not associated, or only weakly associated, with clinicopathologic findings (Supplementary Table S1).

Two polymorphisms were significantly more frequent in female patients: the minor A allele (*p* = 0.039) and the heterozygous GA genotype (*p* = 0.033) of rs17887126 (c.-235G>A), and the minor C allele (*p* = 0.024) and the heterozygous TC genotype (*p* = 0.020) of rs4789551 (c.221+209T>C) (Supplementary Table S1).

Four polymorphisms were significantly associated with smoking: the minor C allele (*p* = 0.017) and CT and CC genotypes (*p* = 0.042) of rs3764383 (c.-1547C>T); the major G allele (*p* = 0.025) and major GG genotype (*p* = 0.020) of rs17887126; the major T allele (*p* = 0.015) and major TT genotype (*p* = 0.012) of rs4789551; and the minor C allele (*p* = 0.032) of rs2239680 (c.*148T>C). The minor genotypes TC and CC of rs2239680 were weakly associated with smoking (*p* = 0.055), as were the major G allele of rs9904341 as well as the minor A allele and CA and AA genotypes of rs2661694 (Supplementary Table S1).

One polymorphism, rs2071214 (c.385G>A), was associated with tumor size. The minor G allele (*p* = 0.018) and the heterozygous GA genotype (*p* = 0.015) only occur in tumors less than 4 cm in size (Supplementary Table S1).

Five polymorphisms were statistically associated with clinical and pathological lymph node (cN and pN) and clinical and pathological TNM (cTNM and pTNM) status, while one (rs17887126) was weakly associated with lymph node status. Three were associated with lymph node status only, and two with both lymph node status and TNM status.

Rs3764383 (c.-1547C>T), rs2239680 (c.*148T>C), and rs2661694 (c.*1373C>A) were statistically associated with pN and cN. The minor C allele (cN *p* = 0.034, pN *p* = 0.029) of rs3764383 was associated with positive lymph nodes, while the CC and CT genotypes were associated with cN (*p* = 0.050). The minor C allele (cN *p* = 0.002, pN *p* = 0.012) and the minor homozygous CC genotype (cN *p* = 0.008, pN *p* = 0.025) of rs2239680 were associated with positive lymph nodes. The minor A allele (cN *p* = 0.023, pN *p* = 0.040) of rs2661694 was associated with positive lymph nodes, while the AA and CA genotypes were associated with cN (*p* = 0.034) and weakly associated with pN (*p* = 0.071).

**Figure 2:**
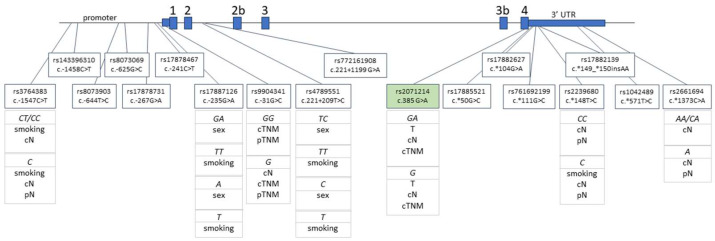
Location of analyzed polymorphisms on the schematic representation of *BIRC5* gene region. Genotypes and alleles of polymorphisms statistically associated with clinicopathological findings are listed. Polymorphism highlighted in green shows a protective effect. The scheme of *BIRC5* contains all annotated exons from the hg38 human genome build. Nucleotide positions are numbered according to NM_001168.2.

Rs9904341 (c.-31G>C) and rs2071214 (c.385G>A) were statistically associated with both lymph node status and TNM status. The major G allele of rs9904341 was associated with a positive cN (*p* = 0.045) and higher cTMN (*p* = 0.004) and pTNM (*p* = 0.006), while the homozygous GG genotype was associated with higher cTNM (*p* = 0.010) and pTNM (*p* = 0.013). The minor G allele of rs2071214 was associated with a negative cN (*p* = 0.011) and lower cTNM (*p* = 0.001) and weakly associated with lower pTNM (*p* = 0.059). The heterozygous GA genotype was associated with a negative cN (*p* = 0.009) and lower cTNM (*p* = 0.0005) and weakly associated with a lower pTNM (*p* = 0.054) (Figure 2).

## 3. Discussion

In this retrospective study, eighteen polymorphisms were found in the *BIRC5* gene region. Fifteen of these were found in HNSCC patients, whereas three were found only in control subjects.

LD has been previously reported in HNSCC [12,15,20,21], but this study showed no significant difference between HNSCC samples and controls.

Eleven polymorphisms that exhibited a MAF > 5% were compared with clinicopathological findings. Four polymorphisms (rs8073903, rs8073069, rs17878467, and rs1042489) showed no or only a weak association with clinicopathological findings. Seven polymorphisms showed a significant association with one or more clinicopathological findings.

The main finding of this study is that five polymorphisms were statistically associated with clinical and pathological lymph node (cN and pN) and TNM status (cTNM and pTNM), while two others were associated with sex and smoking.

For rs3764383 (c.-1547C>T), the minor C allele and the minor CC and CT genotypes showed a significant correlation with positive lymph node status and also with smoking. Although the polymorphism is usually referred to as C>T, in all populations listed in the NCBI SNP database (https://www.ncbi.nlm.nih.gov/snp, last accessed on 3 November 2023), C is the minor allele. In some papers, it is referred to as c.-1547A/G and has not been associated with positive lymph nodes, but has been associated with poorer survival in non-small-cell lung cancer [18] and earlier age of onset in ovarian and breast cancer [19,20]. It has been shown to be associated with susceptibility to bladder cancer [22]. On the other hand, it was not connected with susceptibility to breast [20,23] or liver cancers [24].

Rs17887126 (c.-235G>A) is a relatively rare polymorphism which has not been observed in some Asian populations [15]; even in some European populations, it was too rare to correlate with clinical data [25]. It is located at a binding site for several transcription factors [20,26] but seems not to be connected with high survivin expression in cancers [25,27]. In this study, a slightly higher frequency of the minor A allele and the GA genotype was found in patients. Our group found the same pattern in ovarian cancer [21]. The minor A allele and GA genotype also showed a marginally significant correlation with tumor location (more frequent in the oral cavity), higher Broders status, and negative lymph nodes. They were also significantly more common in women, but the major G allele and the major GG genotype were significantly associated with smoking.

For rs9904341 (c.-31G>C), the major G allele and GG genotype were associated with positive lymph nodes and higher TNM status in this study. This polymorphism is the most studied *BIRC5* polymorphism [28] and the subject of several meta-analyses [29,30,31]. It is located in the 5′UTR region of the *BIRC5* gene and modifies the binding motif of the CDE/CHR repressor [27]. It has been associated with susceptibility (oral [15,32], esophageal [16,33], colorectal [17,34,35], lung [14], and urinary tract cancers [22,31,36,37,38,39,40]), survival (colorectal [17] and lung cancers [41]), and age of onset (ovarian [19] and prostate cancers [42]). As in this study, an association was also found with stage and lymph node status (oral [43,44], esophageal [33], pancreatic [45], urinary [36,38,39], lung [46], and colorectal cancers [34]). On the other hand, although Ma et al. found that the CC genotype was significantly increased in Chinese nasopharyngeal carcinoma patients, they found no significant association with TNM stage [47]. Similarly, Aynaci et al. found that carriers of the heterozygous GC genotype had a lower risk of developing lung cancer; they found no association with tumor stage, lymph node, or metastatic status [48]. Kawata et al. found that although the CC genotype had a significantly higher risk of bladder cancer, no association between this polymorphism with tumor grade or stage was found [37]. Kostić et al. found no difference in allele or genotype frequencies between the patients and controls in oral SCC [49]. Hmeljak et al. found no association between this polymorphism and survival in malignant pleural mesothelioma patients [50]. The data from the literature are very contradictory for this polymorphism, as some papers state that the C allele is associated with pathological factors, while others state that it is G that is associated. This could be explained by the fact that, in some populations, the frequency of both alleles is almost equal (https://www.ncbi.nlm.nih.gov/snp, last accessed on 3 November 2023), and in some articles, it has sometimes been referred to as c.-31C>G [14,36]. In addition, most papers agree that this polymorphism is a better predictor for Asian populations [29,38,51].

Rs4789551 (c.221+209T>C) is a relatively rare polymorphism located in intron 2 of the *BIRC5* gene. In this study, the minor C allele and the heterozygous TC genotype were more common in women, while the major T allele and the major TT genotype were significantly associated with smoking. In the literature, this polymorphism has been associated with poorer survival in non-small-cell lung cancer [18].

For rs2071214 (c.385G>A, also known as c.9194G>A, p.Glu129Lys), the minor G allele and the heterozygous AG genotype were significantly associated with smaller size, negative lymph nodes, and lower TNM status in this study. It is the only polymorphism in this study that is located in the coding region of the *BIRC5* gene and is a missense variant. It also seems to have a protective role. Although this polymorphism is commonly referred to as G>A in all populations listed in the NCBI SNP database (https://www.ncbi.nlm.nih.gov/snp, last accessed on 3 November 2023), G is the minor allele. This polymorphism is usually referred to as c.9194G>A. Although Kawata et al. found no correlation of this polymorphism with tumor grade or stage, they found that the AG and GG genotypes were associated with a significantly lower risk of bladder cancer [37]. On the other hand, the GG and GA genotypes were found to be associated with an increased risk of breast [52] and prostate cancers [42].

For rs2239680 (c.*148T>C, also known as c.9386T>C), the minor C allele and the CC genotype were associated with positive lymph nodes in this study. The minor C allele was also associated with smoking. It is located at the 3′UTR region of the *BIRC5* gene, and Zu et al. found that miR-335 binds in this region, so this polymorphism might change *BIRC5* expression [53]. In most previous publications, this polymorphism was referred to as c.9386T>C. In the literature, the C allele was associated with an increased risk of lung [53] and prostate cancer [42], as well as a higher stage in lung cancer [53]. On the other hand, the major T allele has been associated with an earlier age of onset in breast cancer [20].

For rs2661694 (c.*1373C>A, also known as c.10611C>A), the minor A allele and the AA and CA genotypes were associated with positive lymph nodes in this study. They were also weakly associated with smoking. It has been shown in the literature not to be a good prognostic marker for breast cancer [23], and no statistically significant associations were found for susceptibility to lung [18], ovarian [21], or breast cancer [20].

A literature search found only nine studies analyzing *BIRC5* polymorphisms in head and neck squamous cell carcinomas. Six of those only analyzed the rs9904341 (c.-31G>C) polymorphism [32,33,43,44,47,49], while the other three also analyzed selected polymorphisms in the promoter, coding, and 3′UTR regions [12,15,16]. As far as we know, this is the first study on *BIRC5* polymorphisms in HNSCC patients analyzing the whole coding region of *BIRC5*.

The main limitation of this study is the relatively small number of patients due to the small population size in the analyzed region, which meant that only a limited number of patient samples were available. The sample archives usually contain only the FFPE samples, and the quality of DNA from that type of sample is inadequate for this type of analysis, so only blood DNA samples were used.

## 4. Materials and Methods

### 4.1. Patients and Clinical Samples

For this retrospective study, forty-eight DNA samples collected from HNSCC patients in the Department of Maxillofacial Surgery, University Hospital Osijek, during 2007–2009 were used [54]. The demographic and clinicopathological data of the patients involved in this study are listed in Table 1. All samples were collected according to the ethical principles approved by the Ethics Council of the Osijek Clinical Hospital (No. 29-1:1688-12/2006) and in accordance with the Declaration of Helsinki. Signed informed consent forms were obtained from all patients. The inclusion criteria were adult patients with an HNSCC tumor of any stage. Exclusion criteria were previous HNSCC tumors in the same patient and previous chemo- or radiotherapy. For genotyping, DNA samples were collected from 74 healthy controls with no history of cancer in our previous study [55]. Non-age-matched controls were deliberately used, as this older healthy population (median age 80, range 65–101 years) is less likely to have cancer-predisposing polymorphisms.

### 4.2. SNP Selection and Genotyping

The entire coding region (including the alternative exons 2α, 2B, and 3B) was genotyped, including the six SNPs in the *BIRC5* promoter and four SNPs in the 3′UTR region selected from the National Center for Biotechnology Information SNP database (http://www.ncbi.nlm.nih.gov/snp, last accessed on 3 November 2023). Thirteen PCR fragments (3 in the promoter region, 1 in the 5′UTR region, 7 covering *BIRC5* exons, and 2 in the 3′UTR region) were analyzed using high-resolution melting analysis on the High Resolution Melter (HR-1, Idaho Technology, Salt Lake City, UT, USA), as described in Cvok et al. [55]. Aberrant PCR products were sequenced using the Big Dye Terminator 1.1 Cycle Sequencing Kit (Applied Biosystems, Foster City, CA, USA) and analyzed on an ABI PRISM 310 Genetic Analyzer (Applied Biosystems). Due to the presence of several different polymorphisms in the PCR product of exon 4 and the start of the 3′UTR region, it was directly sequenced. The PCR cycling conditions and primers’ sequences have been published previously [21]. Due to the high GC content in the DNA sequence of the promoter region, CG RICH buffer (Roche, Mannheim, Germany) was added to all PCR fragments located in the promoter as per manufacturers’ instructions.

### 4.3. Statistical Analysis

Differences in allele and genotype frequencies between cases and controls, and association between genotypes, alleles, and clinicopathological variables, were analyzed using Fisher’s exact test (2 × 2) and χ^2^ test (3 × 2). Online SHEsisPlus tool (http://shesisplus.bio-x.cn/SHEsis.html, last accessed on 3 November 2023) [56] was used for assessing deviation from the Hardy–Weinberg equilibrium (HWE), using the χ^2^ goodness-of-fit test, and linkage disequilibrium between polymorphisms, by calculating the squared correlation coefficient (R^2^) between allelic values at two loci. Association between age and alleles, and age and genotypes, was analyzed using Mann–Whitney and Kruskal–Wallis tests, respectively. Survival analysis was performed with the Kaplan–Meier method and survival curves were compared using the log-rank test. Two-tailed *p*-values ≤ 0.05 were considered statistically significant. Statistical analysis was performed using MedCalc v22.014 (MedCalc Software bvba, Ostend, Belgium).

## 5. Conclusions

This study identified a total of 18 polymorphisms in the *BIRC5* gene, 11 of which had a minor allele frequency (MAF) of more than 5% in head and neck squamous cell carcinoma (HNSCC) patients in Croatia. Five of these polymorphisms (rs3764383, rs9904341, rs2071214, rs2239680, and rs2661694) were associated with advanced clinical and pathological lymph node (cN and pN) status. They were also linked to higher TNM stages (both clinical and pathological). The results show that these *BIRC5* polymorphisms do not have a significant impact on patient survival, and shed light on the genetic factors associated with the progression of HNSCC. Interestingly, the only polymorphism detected in the coding region of the *BIRC5* gene, rs2071214, demonstrates a protective role in HNSCC progression.

## Figures and Tables

**Table 1:** Demographic and clinicopathological data of patients.

Characteristic	No. of Patients (%)
Age, years	
median (range)	59 (39–78)
Sex	
male	41 (85.4%)
female	7 (14.6%)
Tobacco consumption	
no	6 (12.5%)
yes	42 (87.5%)
Type	
oral	31 (64.6%)
oropharyngeal	17 (35.4%)
T	
1	3 (6.2%)
2	19 (39.6%)
3	11 (22.9%)
4	15 (31.2%)
cN	
0	20 (41.7%)
1	11 (22.9%)
2	15 (31.2%)
3	2 (4.2%)
cTNM	
I	2 (4.2%)
II	10 (20.8%)
III	11 (22.9%)
IV	25 (52.1%)
pN	
0	16 (33.3%)
1	5 (10.4%)
2	25 (52.1%)
3	2 (4.2%)
pTNM	
I	3 (6.2%)
II	7 (14.6%)
III	7 (14.6%)
IV	31 (64.6%)
Broders	
1	19 (39.6%)
2	22 (45.8%)
3	5 (10.4%)
4	2 (4.2%)
Survival	
alive	8 (16.7%)
deceased	35 (72.9%)
N.A.	5 (10.4%)

T—tumor size, cTNM—clinical staging system, cN—clinical regional nodal status, pTNM—pathological staging system, pN—pathological regional nodal status, Broders—histopathological grading. N.A.—data not available.

**Table 2:** Polymorphisms in the *BIRC5* region found in this study. Nucleotide positions are numbered according to NM_001168.2. Bracketed numbers are legacy notations commonly used in the literature.

Gene Region	SNP ID Number	Nucleotide Change	Minor Allele Frequency Controls (*n*/N, %)	Minor Allele Frequency OSCC Cases (*n*/N, %)	*p*-Value (for Genotype Frequencies)	*p*-Value (for Allele Frequencies)
promoter	rs3764383	c.-1547C>T *	37/148 (25.0)	26/92 (28.3)	0.327	0.651
promoter	rs143396310	c.-1458C>T	2/148 (1.3)	0/92 (0.0)	0.523	0.525
promoter	rs8073903	c.-644T>C	49/148 (33.1)	35/96 (36.5)	0.548	0.679
promoter	rs8073069	c.-625G>C	33/148 (22.3)	21/96 (21.9)	0.977	1.000
promoter	rs17878731	c.-267G>A	1/148 (0.7)	0/96 (0.0)	1.000	1.000
promoter	rs17878467	c.-241C>T	16/148 (10.8)	12/96 (12.5)	0.666	0.686
promoter	rs17887126	c.-235G>A	2/148 (1.4)	6/96 (6.3)	0.056	0.060
5′UTR	rs9904341	c.-31G>C	55/148 (37.2)	30/92 (32.6)	0.581	0.491
intron 2	rs4789551	c.221+209T>C	7/148 (4.7)	5/92 (5.4)	1.000	0.772
intron 2	rs772161908	c.221+1199G>A	0/148 (0.0)	1/96 (1.0)	0.393	0.393
exon 4	rs2071214	c.385G>A ** (c.9194G>A)	5/148 (3.4)	5/96 (5.2)	0.513	0.521
3′UTR	rs17885521	c.*50G>C (c.9288G>C)	3/148 (2.0)	1/96 (1.0)	1.000	1.000
3′UTR	rs17882627	c.*104G>A (c.9342G>A)	2/148 (1.3)	0/96 (0.0)	0.519	0.522
3′UTR	rs761692199	c.*111G>C, (c.9349G>C)	0/148 (0.0)	1/96 (1.0)	0.393	0.393
3′UTR	rs2239680	c.*148T>C (c.9386T>C)	34/148 (23.0)	25/96 (26.0)	0.824	0.647
3′UTR	rs17882139	c.*149_*150insAA, (c.9387_9388insAA)	3/148 (3.8)	1/96 (1.0)	1.000	1.000
3′UTR	rs1042489	c.*571T>C (c.9809T>C)	53/148 (35.8)	38/96 (39.6)	0.642	0.589
3′UTR	rs2661694	c.*1373C>A (c.10611C>A)	38/148 (25.7)	21/96 (21.9)	0.642	0.543

* T is a major allele in Croatian population. ** A is a major allele in Croatian population.

## Data Availability

Data is contained within the article and Supplementary Materials.

## Supplementary Materials

The following supporting information can be downloaded at: https://www.mdpi.com/article/10.3390/ijms242417490/s1.
